# Deontic signs increase control monitoring: evidence from a modified traffic flanker task

**DOI:** 10.1007/s10339-023-01139-z

**Published:** 2023-04-28

**Authors:** Teresa Garcia-Marques, Pedro Figueira, Alexandre Fernandes, João Martins

**Affiliations:** William James Center for Research, ISPA - Instituto Universitário, Rua Jardim do Tabaco, 34, 1149-041 Lisbon, Portugal

**Keywords:** Traffic signs, Deontic norms, Flanker task, Cognitive control

## Abstract

Deontic norms are expected to impose individuals’ control over their behavior. In this paper, we address such norms presented in traffic signs and test their influence over executive control functions. For Experiment 1, we develop a traffic flanker task in which the typical neutral arrows are replaced with traffic prohibition/obligation signs. Experiment 2 isolated the deontic aspect of the signs using simple arrows on red, blue, and green backgrounds and either primed them to be interpreted as traffic signs or as elements of a gaming console controller. Results in both studies show evidence of controlling context interferences more efficiently when dealing with deontic (traffic) signs than with simple arrows (Experiment 1) or with similar perceptive targets when primed with a deontic context than with a gaming context (Experiment 2). In both studies, obligation/blue signs mitigate flanker effects less than prohibition/red signs. Stimuli color affects the alertness of the cognitive system, with the color red being, by itself, a cue for increased control. Based on temporal analysis, we further discuss these results as evidence of an increase in proactive control that aims to prevent the occurrence of undesirable influence.

## Introduction

The social norms of prohibition and obligation are communicated through different types of signs, which can be linguistic, or symbols of different kinds. For instance, we can read the words “Danger” or “Prohibited” on a door, a word saying “Exit” in a corridor, an arrow signaling a direction, or a simple color suggesting to stop or advance. These signs are not only likely culturally learned, but also possibly grounded in our psychophysiology and perceptive features (e.g., shapes and colors) and seem to communicate deontic injunctive norms that aim to mandate or prevent certain behaviors. This instigation is likely directly related to an increased need to exert cognitive control over people’s actions.

People’s understanding of assertions about rights and duties are made within different shared mental schemas (e.g., Cheng and Holyoak 1985). Children seem to develop these shared mental representations, showing different deontic abilities very early in their development (e.g., Cummins 1996); deontic norms are usually well-attended to, and their processing facilitates the detection of any of its violations (e.g., Cosmides 1989). The dynamic interplay becomes complete when our society uses deontic signs to communicate such social norms (e.g., to define which behavior society expects from an individual) as instruments designed to modulate individuals’ reactions (see Hilpinen 1981; Manktelow and Over 1991).

However, research has not yet fully addressed how deontic norms relate to cognitive monitoring or cognitive control mechanisms that sustain a coherent reaction. Research regarding deontic norms (see Cummins 1996, Johnson-Laird et al. submitted; Oaksford and Chater 1996 for reviews) has mainly focused on the understanding of how well people perform in deontic versus other reasoning tasks, focusing both on how these rules are mentally represented, and on how people develop these deontic capabilities (see Beller 2010). In this regard, the mental model approach informs us that deontic reasoning is anchored in how a situation is represented and how premises are generated from that representation, reflecting the expected reactions in those contexts (e.g., Castro et al 2008; Manktelow and Over 1995; Vargas et al 2011). But these studies on deontological reasoning (an example of high-level cognition), while highly relevant to our understanding of the deontological logical mind, do not inform us about the cognitive control mechanisms that underpin the conformal behavior.

There is, however, one approach that can tell us something about the possibility of deontic contexts interfering with cognitive control mechanisms: research on the role played by color in communicating deontic norms. This is because the color red (at least in our society) is assumed to be either directly or indirectly perceived as a signal of prohibition (e.g., red traffic lights, stop signs). Evidence supporting this relationship shows that red interferes with the processing of words with congruent (facilitating) and incongruent (inhibiting) deontic meanings (Mehta and Zhu 2009). Further evidence shows that this color is likely increasing cognitive control, by showing that a red background increases cognitive vigilance (e.g., Wang et al. 2016) and enhances performance on a detail-oriented task (Mehta and Zhu 2009). Likewise, the hypothesis of increased cognitive control being exerted when the color red is added to the background of an interference task was supported in Wang et al.’s study (2016). However, red was also shown to mean danger, relate to failure in achievements, and evoke a motivated state of avoidance (e.g., Elliot et al. 2007). These associations open the results we reviewed to alternative interpretations. Because of that the question of whether more cognitive control is promoted within a deontic context is still an open question.

In this paper, we address whether a deontic context increases individuals’ monitoring mechanisms, mapped in a typical cognitive interference task (a flanker task). A cognitive interference task is likely to isolate individuals’ attentional capacities to inhibit undesirable interferences, which allows us to address the importance of the deontic context when the deontic feature is not relevant to the task. To do that, we relied on a specific deontic context: the prototypical context of traffic signs (see, for instance, Castro et al. 2008).

Traffic signs are an effective way to communicate a social norm. Although not all traffic signs are normative—by communicating, for instance, the entrance to a specific zone or city—most of them are. These normative signs inform us which norms are expected to be followed by all members of society, such as ensuring drivers’ safety through their predictable, efficient, and orderly movement. Good examples of deontic normative signs are obligation and prohibition signs. Their goal is to directly and efficiently communicate the deontic norm. As such, they were designed to maximize the quick apprehension of the norm, with perceptive features that modulate our attention through variations in shape and color (dominant visual features; see Johansson and Rumar 1966; Greenhalgh and Mirmehdi 2012). Although colors are used to favor the detection and the recognition of traffic signs, they, by themselves, also convey the deontic norm of obligation (using the color blue) or of prohibition (using the color red). Therefore, traffic signs are prototypical deontic signs, designed to impose different levels of alertness, to drive our attention, and to direct our behavior.

In this research, we use traffic signs to convey a deontic norm, expecting them to activate a monitoring mindset that facilitates the inhibition of undesirable interferences, independently of it being an obligation (blue) or a prohibition (red) sign. This will attest that within a deontological context, our minds are driven to activate the necessary control mechanisms to ensure the desirable outcome.

### Overview of research

Two experiments will address the hypothesis that a deontic context will prompt increased monitoring. We use traffic signs to prime the deontic feature of the context and a flanker task to directly assess individuals’ ability to control undesirable interferences in their behavior. We use two deontic contexts—prohibition and obligation—and assess levels of flanker interference as a measure of failure of control.

Both experiments rely on a modified version of the flanker task paradigm. In a flanker task, participants are asked to respond to a central target, flanked by a set of distractors that are known to activate either a congruent or incongruent response (Eriksen and Eriksen 1974). In the classic versions, the congruent and incongruent trials are created by using simple arrows as both targets and flankers, either pointing in the same direction (congruent trials) or pointing in opposing directions (incongruent trials). The modified versions used in our experiments replaced the simple arrows either by traffic signs (Experiment 1) or arrows centered in a circle with a background color (green, blue, or red: Experiment 2).

Performance on a flanker task provides information regarding the control individuals exert over their performance. Interference exerted by the context in a flanker task is evidenced by slower and less accurate responses to targets flanked by incongruent stimuli when compared to responses to targets shown with congruent flankers: flanker effects (represented by a delta index; e.g., Eriksen and Schultz 1979; Eriksen and St. James 1986; Stoffer 1991). The dynamic nature of control activation can be mapped in this task by plotting the delta function (the magnitude of the effect at different response times; see Ridderinkhof 2002). The flanker effects tend to increase with an increase in response times. This leads the delta plots function, over the individual mean response times, to have a positive slope (e.g., Davranche et al. 2009). As such, the evidence of a monitoring mindset imposed by a deontic context may be found in the context leading to an immediate and consistent increase in control over the undesirable influence. In this case, if control becomes efficient, the delta function will not show a typical increase in their slopes, attesting that control is exerted proactively (see Botvinick et al. 2001). Alternatively, contrary to what is expected by the hypothesis of control being promoted by the deontic nature of the context, slopes can show evidence of reactive control—that is, evidence that control is exerted over the detection of interference. In this case, the delta function slopes will be expected to reduce with increasing response time (see Burle et al. 2014).

Our first approach to the hypothesis (Experiment 1) tested whether obligation (blue) and prohibition (red) traffic signs affected participants’ performance in a flanker task, compared with a classic flanker task, showing evidence of proactive control (akin to increased monitoring promoted by the context). In Experiment 2, we control for a color effect contrasting data obtained with the same colors of the deontic context but within a non-deontic setting.

We expect participants to perform better (showing evidence of higher control) in a traffic flanker task than in classic flanker task (henceforth referred to as the *neutral* task), given the deontic nature of the first. Differences between the level of control exerted in the obligation and the prohibition context may emerge, given the previous evidence suggesting that the color red promotes a better overcoming of the conflict than the color blue (Wang et al. 2016). This color effect will be better understood in Experiment 2.

In Experiment 1, we take several methodological precautions: (1) we pre-test the deontic meaning of our materials, to be sure that a deontic component was being activated when participants were performing a task; (2) the neutral flanker (classic flanker) is always presented as the first task, to prevent it from being influenced by a normative mindset, previously activated by the traffic signs, and (3) we counterbalance the order of the prohibition and obligation flanker tasks to isolate the possible influence of the color red over the color blue, and thus not confound the color and deontic context effects. We also add a social context factor to our design (the hypothesis was tested in a co-action and isolated conditions) to control for the fact that traffic signs are social norms. This will inform whether the activation of those norms is modulated by the social context, given that control is already known to be higher in others’ presence (Fernandes et al. 2019; see Belletier et al. 2019 for a review).

In Experiment 2, we clarify possible color effects, testing the impact of signs of equal color both inside and outside a deontic context. As such, in this experiment, we replace the traffic signs in the flanker task with simple arrows superimposed on a blue, red, or green background, and manipulate the nature of the context through priming—either by previously associating colors with traffic signs or with a gaming console controller (where colors offer different decisional opportunities of reacting, instead of a societal demand for a specific reaction). Once again, we assumed that participants would perform better in the deontic context (traffic) than in the non-deontic context (game), suggesting that the deontic context is influencing their monitoring abilities. Given the reviewed literature, we also expect to find evidence of higher control when the background is red.

## Experiment 1

### Participants and design

Participants were 88 university students (30 men; *M*_age_ = 22.58; SD_age_ = 7.56), who obtained partial course credit through their participation.

All participants performed a neutral flanker task, followed by two traffic flanker tasks, being randomly distributed between the two counterbalanced orders of the prohibition and obligation tasks. Half of the participants performed these tasks alone, the other half in co-action. G*Power (Faul et al. 2007) analysis shows our sample size (> 74) as adequate for the identification of within and between effects and their qualification by the two groups, with a size of *f* = 0.25, *α* = .05, and a power of .90.

### Materials

#### Traffic signs

Prohibition and obligations traffic signs, with arrows pointing to the right or to the left, were selected for Experiment 1. A pilot study (*N* = 116 participants with a driver’s license; 60 male; *M*_age_ = 23.20; SD_age_ = 6.68) accessed the likelihood of prohibition and obligation norms being activated by each of these signs. Participants were briefly presented with either a “mandatory left turn” sign (obligation) or a “no left turn” sign (prohibition) and asked to, as quickly as possible, write the first three words that came to mind when presented with the sign. Results show that when faced with a prohibition sign 95% of participants mention either the word “prohibition” (42%), or the equivalent statement, “do not” (53%). When shown an obligation sign, 56.4% of the participants provide a word semantically related to “obligation,” and 43.6% classified either the direction of the arrow or the stimulus as a traffic sign.

#### Flanker tasks

**Fig. 1:**
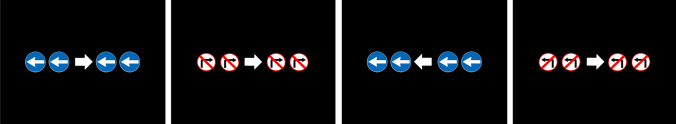
Incongruent and congruent trials of the obligation flanker task and the prohibition flanker task

Three different versions of the flanker task are used in our experiment. A neutral flanker using simple arrows pointing in one of two directions, right and left, and two modified versions that replaced the simple arrows with either an obligation or a prohibition sign. Both for simple arrows and traffics sign arrows, four types of five-arrow strings were used to define the incongruent and the congruent trials. These differ in that the direction of the middle arrow is either in the same or in the opposite direction of its flankers (see Fig. 1).

### Procedure

By enrolling in the experiment, participants signed an informed consent form for their participation in the study. Experimental sessions were scheduled in groups or individually, to manipulate social context. In the *isolation* condition, each participant was left alone in a cubicle in front of a computer screen where all instructions and stimuli were presented using the *E-Prime* 2.0 software. In the *presence of others* condition, participants arrived in groups of 6 to 8 participants, sat in front of a computer in individual contiguous booths, and were asked to start their participation simultaneously (co-action).

All participants were first familiarized with the flanker instructions and materials, performing 12 practice trials (six congruent and six incongruent) before engaging in the experimental tasks. The first task was the neutral flanker task, followed by one of the two traffic flanker tasks that were presented in a counterbalanced order. Half of the participants were randomly distributed to the condition in which they executed the prohibition task first, and the other half to the condition in which they executed the obligation task first. Instructions for each flanker task asked participants to identify the direction that the middle arrow on the screen was pointing to, left or right, while ignoring the flanking stimuli. Participants used the keyboard arrows to respond.

Each flanker task was composed of five blocks of 20 trials each (ten incongruent, ten congruent). Each trial showed a fixation screen (500 ms), followed by the target stimuli. (Participants had 1500 ms to respond.) A black screen of 1500 ms separated different trials. Reaction times and response accuracy were measured in each trial.

In the end, participants were thanked for their participation and offered a link to access a debriefing if they desired.

### Results and discussion

Interference scores (incongruent–congruent trials) for each flanker task type were calculated for the proportion of correct responses (CRs) and reaction times (RTs) of the CRs. Data regarding abnormally high levels of interference—more than 3 SDs above the general mean (three outliers) and very short RTs (< 200 ms; six outliers)—were a priori excluded from the analysis.

#### Interference within the flanker task types

Interference scores of each flanker task type were entered as a within-subject factor (neutral, prohibition, and obligation) into a two-factor ANOVA, with social presence as a between-subject factor.

**Fig. 2:**
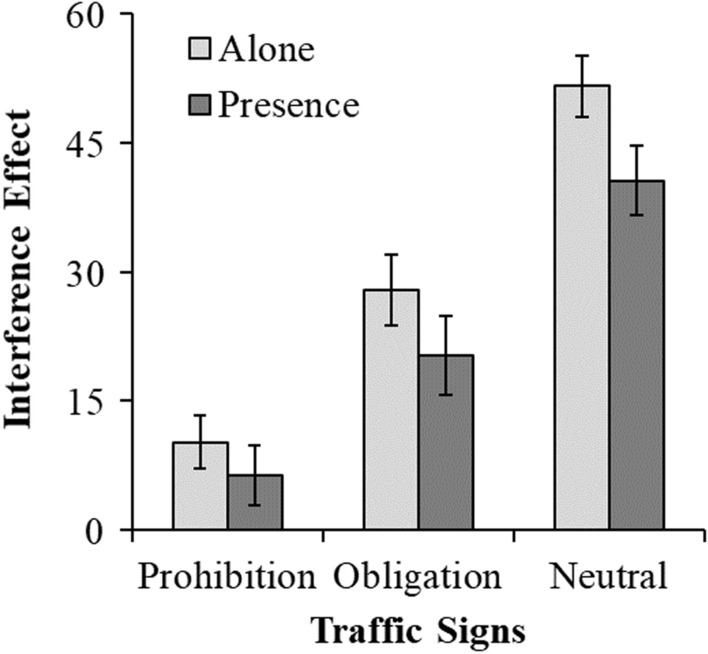
Interference-RT scores (RT_incongruent_ − RT_congruent trials_) in milliseconds for each flanker task (Experiment 1)

The analysis of the interference-RT scores shows the expected main effect of flanker task type as significant, *F*(2, 154) = 52.93, *p* < .001; *η*^2^ = .41, such that (a) interference was reduced for both traffic flanker task types relative to the neutral task (contrast weights: − 1, − 1, 2; *Tukey: t(*77) = 10.25, *p* < .001; *d* = 2.34) and that the prohibition task led to less interference than the obligation task (contrast weights − 1, 1, 0; *Tukey*: *t*(77) = 3.85, *p* < .001*; d* = 0.88). As also expected, less interference occurred in the presence of others (*M* = 22.40; SD = 2.55) relative to participants in the isolation condition (*M* = 29.88, SD = 2.27), *F*(1, 77) = 4.78, *p* = .032; *η*^2^ = 0.06). The non-significant interaction, *F*(2, 154) = 0.47, *p* = .624, suggests, however, that the flanker task type effect was similar in these two social conditions (see Fig. 2).

The analysis of the levels of interference in proportion of CRs show them to be generally close to zero. Even so, a significant main effect of flanker task type, *F*(2, 154) = 6.05, *p* < .003, *η*^2^ = 0.07, was detected: interference affected the nature of the response more in the neutral flanker task (*M* = .02; SD = .002) than in the two traffic flankers (obligation: *M* = .00, SD = .003; prohibition: *M* = .01, SD = .003). No difference between the two traffic flankers was detected (*t* < 1). There was no evidence of a social presence effect, *F*(1, 77) = 0.60, *p* = .440, nor of a social presence moderation of the type of flanker effect, *F*(2, 154) = 0.30, *p* = .736.

Neither order of the traffic flanker task types nor participant gender moderated any of the described effects regarding interference-RT and interference-CR (all *ps* > .60).

#### Temporal distribution analysis: interference as delta plots

To analyze the temporal dynamics of the control exerted in each flanker task, correct response RTs were grouped by congruence condition (congruent and incongruent), then rank-ordered and divided into five RT quantiles (*bin*s) of equal or near-equal size. Only data from participants with more than 80% of correct responses to incongruent trials could be included in this analysis. By subtracting the quantile RT mean for incongruent trials from the RT mean for congruent trials, we calculated the delta values and plotted them against the cumulative probabilities, such that each point in the delta plot was represented by *yi* = [(bin-*i*_incongruent_) − (bin-*i*_congruent_)] and *xi* = [(bin-*i*_incongruent_) + (bin-*i*_congruent_)]/2 (Ridderinkhof 2002). Level of interference in each *bin* was then analyzed within an ANOVA, with *bins* and type of flanker tasks as within factors and social presence as a between factor.

**Fig. 3:**
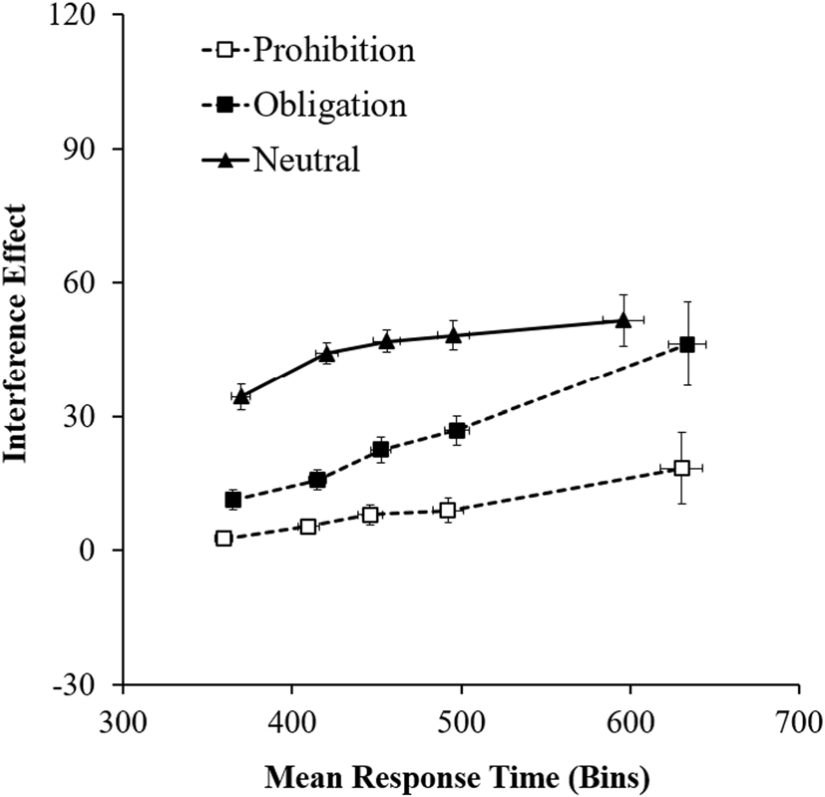
Interference temporal distribution in milliseconds for each type of flanker task (Experiment 1)

This analysis identified the previously detected flanker effects as also showing the expected bin effect, *F*(4, 320) = 4.69, *p* < .001, *η*^2^ = 0.06, since the magnitude of the interference observed in each trial is dependent on the time participants takes to respond to that trial. Importantly, while it was not moderated by social presence, *F*(4, 320) = 1.09, *p* = .358, the *bin* effect was moderated by the type of flanker task performed, *F*(8, 640) = 3.82, *p* < .001, *η*^2^ = 0.05 (see Fig. 3). The function showing the typical general positive slope across time (e.g., Davranche et al. 2009) has different patterns for each flanker task type. This suggests that in addition to differences in the intercepts (levels of control), there may also be differences in how control is exerted in each flanker task type, reflected in their slopes.

The slope analysis (see Table 1) was performed to clarify how control was exerted in each flanker task type, with slopes calculated at *an individual level*; for each individual, two consecutive bins were compared (segments connecting Bins 1–2, Bins 2–3, Bins 3–4, Bins 4–5). Positive slopes suggest no control being exerted over levels of interference. The absence of difference between slopes suggests that the ability to overcome the influence is not increasing with response time. Negative slopes suggest that it took participants more time to be able to exert more efficient control over their responses, suggesting reactive control (Ridderinkhof 2002). The comparison between the means of these slopes was made through an ANOVA. Results show the main effect of flanker task type, with slopes being generally higher for the prohibition flanker and lower for the neutral flanker, *F*(2, 170) = 4.35, *p* = .014, *η*^2^ = 0.05. The interaction with the *bin* factor was also significant, *F*(3, 255) = 2.90, *p* = .035, *η*^2^ = 0.03, and the observed pattern of the means (see Table 1) suggests that time is relevant for the level of exerted control only in the neutral task. In support of this interpretation, the study of the linearity of the curve shows that the slopes only differ for the neutral task (*β* =  − 0.60; *t*(85) =  − 3.20, *p* > .001, *d* = 0.69.

**Table 1:** Delta plots slopes for each type of flanker task (Experiment 1)

Traffic signs	Slopes							
	S1		S2		S3		S4	
Prohibition	0.03	(0.03)	0.06	(0.04)	0.03	(0.04)	0.07	(0.05)
Obligation	0.12	(0.04)	0.20	(0.04)	0.09	(0.04)	0.14	(0.06)
Neutral	0.20	(0.03)	0.08	(0.04)	0.03	(0.04)	0.01	(0.05)

#### Conflict adaptation across performance

Because practice allows participants to perform better on a flanker task, likely by a gradual narrowing of attentional focus on the target item (e.g., Heitz and Engle 2007), we should expect it to interfere with differences between the different types of flanker tasks. This may have contributed to the better performance in the traffic flanker tasks than in the neutral one, given that they were performed last. This “practice” in overcoming interference results in a “conflict adaptation.” Although this needs to be tested further (see Experiment 2), there are some features of our data that are informative regarding the likelihood of this possible confounding. The first feature is the lack of order effects; the pattern of our results suggests no order effects, such that the flanker effects for the obligation and prohibition tasks were similar whether one was presented before or after the other. The second feature is the pattern of conflict adaptation that occurred within each flanker task (across their five blocks). The ANOVA, with the five blocks as a new factor, besides replicating previously identified effects, shows only its main effect as significant, *F*(4, 320) = 4.22, *p* = .002; *η*^2^ = 0.05. No interactions were significant (*F* < 1). However, contrary to the conflict adaptation expectation, we find no linear increase in efficiency with time, *t*(81) = 1.19, *p* = .236. Instead, a significant curvilinear trend, *t*(81) = 3.62, *p* < .001, *d* = 0.80, suggests that the interference becomes consistently smaller in the middle of each task’s block (interference means in msec: 34, 23, 14, 24, 27).

In sum, overall, Experiment 1 data support our expectation that the level of control activated in a deontic setting differs from the level of control activated in a non-deontic setting. Flanker effects (interference scores) are reduced when the setting has either prohibition or obligation signs, independently of their order. In addition, control is higher for the flanker task types that use the prohibition than the obligation sign. Null differences between the prohibition and obligation tasks at the level of the proportion of correct responses suggest, however, that participants were able to overcome the interference. Further analyses suggest that the deontic context is more likely associated with a pattern of results consistent with what is expected if proactive rather than reactive control occurs.

To better separate the effects of the deontic context from the effects of colors used in the prohibition and obligation signs, we ran Experiment 2. In it, participants were presented with three flanker task types using simple arrows superimposed on circles with different background colors (red, blue, green), either in a deontic or non-deontic setting (experimental priming conditions). We expect, once more, that participants perform better in the deontic context (traffic) than in the non-deontic context (game), suggesting that the first is influencing their monitoring abilities.

Besides this, two other differences were introduced. First, because the effect was not moderated by the presence of others, we use only the *presence* setting; second, we did not focus on a neutral flanker task, but introduced a new flanker with the same type of stimuli on the color green, which is always performed last.

## Experiment 2

### Participants and design

Participants were 110 undergraduate students (87 female; *M*_age_ = 20.1 SD_age_ = 2.44) with age below 35, who obtained partial course credit through their participation.

Participants were randomly distributed into one condition of the *between* factors of the design, 2 (*Priming conditions*: Non-deontic vs. deontic) × 2 (counterbalanced orders of red and blue flanker tasks), performing all the three color flanker tasks (*Type of flanker task*: red, blue, green). Sample size guarantees the detection of a between-participant main effect of priming and the focused between vs. within factor interaction with a medium effect size associated with an *α* = .05 and *power* = .95 (Faul et al. 2007).

### Materials

#### Stimuli

**Fig. 4:**
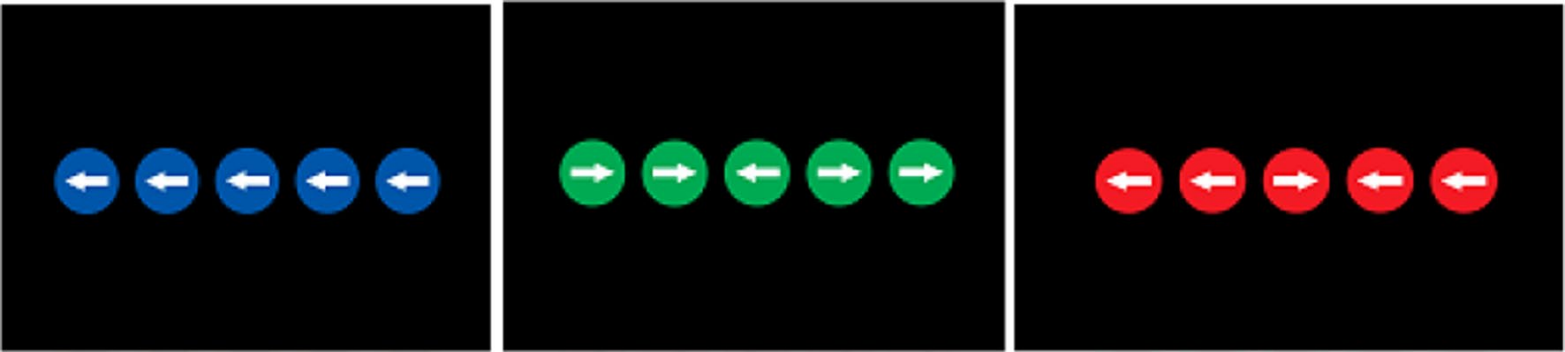
Examples of incongruent and congruent trials for a different types of flanker tasks

The arrows in the strings of arrows that defined congruent and incongruent trials were all inserted in a circle with a background color that was either green, blue, or red, dependent upon the specific flanker task performed (see Fig. 4).

#### Priming instructions

We developed a set of specific instructions to prime either a deontic or a non-deontic perception of the stimuli to be used in the flanker task. A deontic context was created by informing participants that they would perform a task with images simulating traffic signs; several examples of traffic signs were shown on the instruction screen. The non-deontic context was created by informing participants that they would perform a task with images simulating buttons from a gaming console’s controller, and showing the image of a gaming console controller on the instruction screen.

### Procedure

Participants arrived in groups of 6 to 8 to the laboratory and, after reading and signing an informed consent form, were seated in individual open booths, in front of a computer. Participants read experimental instructions similar to those presented in Experiment 1 on the computer screen, managed by the *E-prime* software.

After typing in general demographic information (gender, age), every participant did 20 trials of a neutral flanker task to become familiarized with the experimental setting. Participants were then presented with the experimental task, receiving either the instructions of the *traffic* condition or of the *game* condition, and performed the three flanker tasks. Half of the participants of each experimental priming condition performed the tasks in the red–blue–green order, and the other half in the blue–red–green order. Each task was composed of five blocks of 40 trials (20 incongruent, 20 congruent). Each trial had a fixation screen (500 ms) and a target screen (participants had 1500 ms to respond). A black screen (1500 ms) separated different trials. Reaction times and response accuracy were measured for each trial.

As control measures, participants reported how much the images they had just seen reminded them “1—More of a gaming console controller” or “9—More a traffic sign” and, finally, their familiarity both with gaming console controllers and traffic signs (1—Not at all familiar; 9—Very familiar).

In the end, participants were thanked for their participation and offered a link to access a debriefing if they desired.

### Results and discussion

Data analysis followed the exact procedures used in Experiment 1.

#### Interference within different flanker task types

Reaction time interference scores (incongruent–congruent trials) of each type of task (red, blue, green) were entered as a within-subject factor into a three-factor ANOVA, with the experimental priming conditions and order of the red–blue flanker tasks as between-subject factors.

In favor of our prediction, results show that the magnitude of the flanker effect differs between experimental priming conditions *F*(1, 106) = 4.95, *p* = .028; *η*^2^ = .045. The interference was smaller for the traffic (*M* = 21.3) than for the game (*M* = 41.7) priming condition. When the type of flanker task is considered, a marginal difference emerged, *F*(2, 212) = 3.15 *p* = .063; *η*^2^ = .03. The contrast (Tukey) between the red and blue flankers, *t*(212) = 2.20 *p* = .030; *d* = .30, reliably shows a color red effect. Interference was smaller for the red flanker task (*M* = 27.2) than for the blue flanker task (*M* = 36.3) and is also small for the green flanker task (*M* = 28.9).

**Fig. 5:**
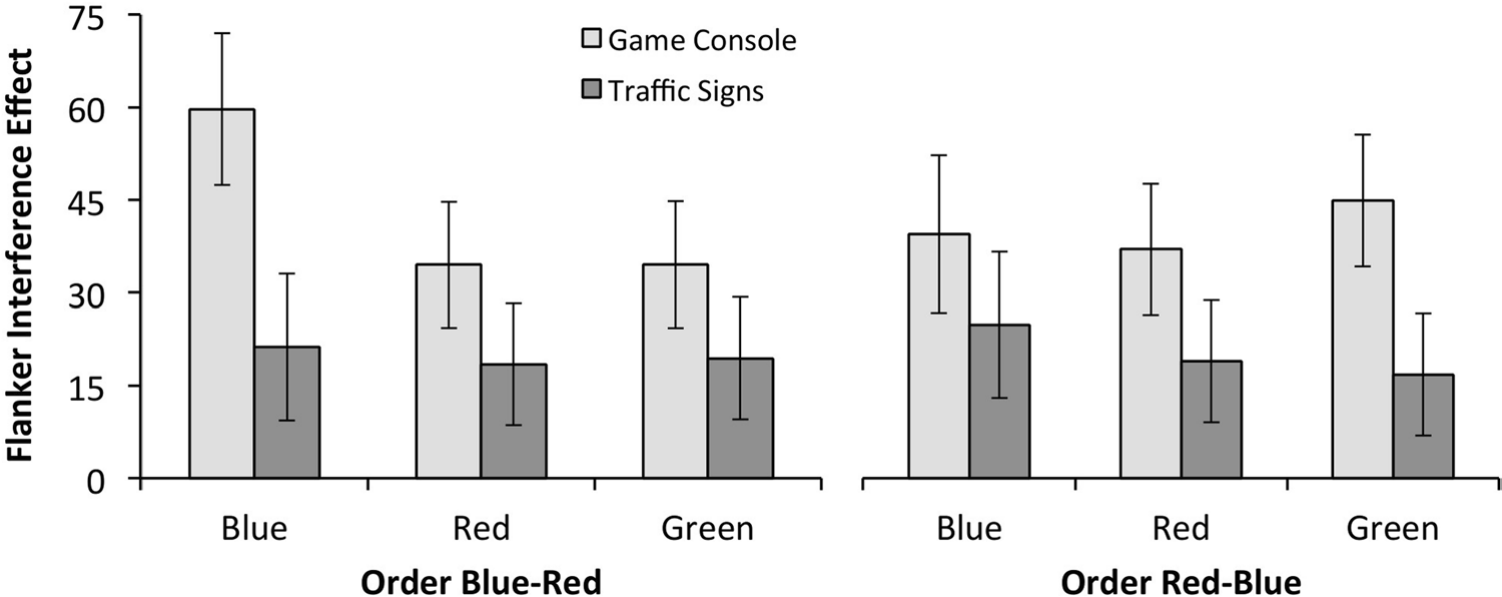
Interference-RT scores (RT_incongruent_ − RT_congruent trials_) in milliseconds for each flanker task (Experiment 2)

No other effect in the analysis was significant. The experimental priming condition x type of flanker task interaction was not significant (*F* < 1), as were all order effects (main effect and interaction; *F* < 1). The three-way interaction was marginal, *F*(2, 212) = 2.66, *p* = .072; *η*^2^ = .03, possibly suggesting differences between the magnitude of the flanker effect found for the game and traffic experimental priming conditions, when the red flanker task was performed first (see Fig. 5).

No significant effects were found for interference in the accuracy of responses (all Fs < 1).

#### Temporal distribution analysis: interference as delta plots

**Fig. 6:**
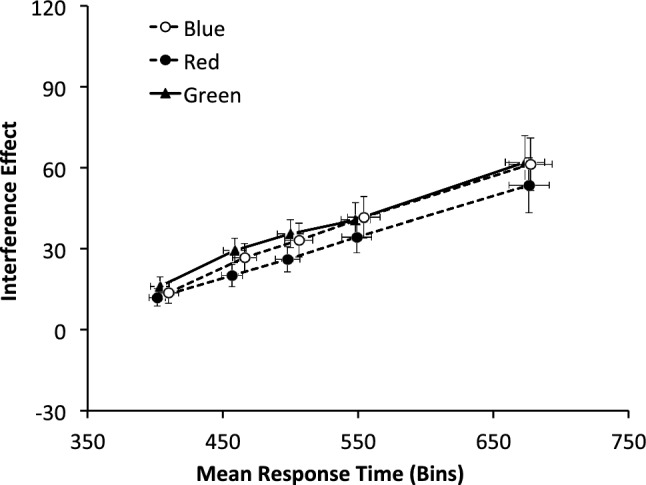
Interference temporal distribution in milliseconds for each task (Experiment 2)

As in Experiment 1, flanker RT-interference effects, given by the differences in RT for congruent and incongruent trials, were separated for each quintile of the RT distribution. Only data from participants with more than 80% of correct responses to incongruent trials could be included in this analysis. The distribution of the flanker effects for each of the five bins was then analyzed for the three tasks, with a repeated measures ANOVA having two within and two between factors in the design. The main effect of *bins* shows some evidence of the usual positive linear trend found for flanker task types, *F*(4, 424) = 36.29, *p* < .001, *η*^2^ = .26 (see Fig. 6). These effects were not qualified by any of the different experimental factors; besides the interaction Bins × Condition × Order, *F*(4, 424) = 1.02, *p* = .398 all other effects had *F* < 1.

The nonsignificant interactions suggest no difference in how interference increases with the delay of a response, which was equal for all types of flanker task. Thus, the higher level of control previously shown to be exerted in the deontic condition occurs independently of response time. This intercept effect suggests that control is implemented immediately (proactively). To test this hypothesis, we analyzed the individual slopes of the delta functions. This slope analysis (see Table 2) shows that they are equal across response times and equal for the three flanker task types (all Fs < 1). With all slopes being positive, we find no evidence of reactive control being instigated by levels of interference (Table 3).

**Table 2:** Delta plots slopes for each type of flanker task (Experiment 2)

	Slopes							
	S1		S2		S3		S4	
Blue	0.18	(0.04)	0.12	(0.03)	0.14	(0.03)	0.14	(0.04)
Red	0.10	(0.03)	0.13	(0.03)	0.13	(0.04)	0.13	(0.05)
Green	0.18	(0.03)	0.10	(0.04)	0.09	(0.04)	0.09	(0.05)

**Table 3:** Delta plots slopes for each experimental priming condition

	Slopes							
	S1		S2		S3		S4	
Game Console	0.17	(0.04)	0.15	(0.03)	0.14	(0.03)	0.12	(0.04)
Traffic Signs	0.14	(0.03)	0.08	(0.03)	0.09	(0.03)	0.12	(0.03)

#### Conflict adaptation across performance

The reduced flanker effects, generally observed with the green background, are likely occurring because those trials were always the last to be executed and were more likely documenting the levels of adaptation to the task. However, when we analyzed levels of interference in participants’ performance across time, with the five blocks of each flanker task type as a factor in the ANOVA (representing the full design), we find no effect of time (*F* < 1) on participants’ performance, suggesting that performance within each flanker task type was not improving with time.

## General discussion

We aimed to test if traffic signs, by conveying deontic social norms, modulate the cognitive control process. To address this question, we focused on one of the most prototypical deontic contexts of our society: traffic signs. In Experiment 1, we use traffic signs of obligation and prohibition as stimuli in a modified flanker task. Responses on these tasks show evidence of the typical flanker effect, with slower RTs when a target arrow was flanked by incongruent arrows, as opposed to congruent (in the same direction) arrows. However, according to the hypothesis that a deontic context primes higher levels of control, participants’ responses were different in traffic flanker tasks relative to a task that uses simple arrows. Both deontic contexts, but more accentuatedly in the prohibition context, show evidence more akin to an assumption of proactive than of reactive control. Results of Experiment 2 help us understand that a stronger control exerted over interference in a prohibition flanker task occurs both because the traffic context, being deontic, increases monitoring of responses, and because the color red, associated with the prohibition sign, is also a cue for vigilance. As such, performance on a flanker task with the red background also shows evidence of increased control over interference in the non-deontic context.

Delta plot analysis (e.g., Forstmann et al. 2008) corroborates previous data (e.g., Davranche et al. 2009) showing that flanker interference tends to increase with an increase in response time, and shows that this occurs for all types of flanker task used in these experiments. This suggests that interference is occurring for all flanker task types, and that no task type imposes total isolation from the context. However, the intercepts of those functions differed, being smaller for the deontic tasks. In addition, slope analysis shows no evidence of negative slopes which would suggest vigilance over the interference being activated and reactivity to it. The zero difference between the slopes that characterize the delta functions suggests that deontic contexts are more efficiently processed by prompting proactive control. Thus, the delta plot analysis of both experiments sustains our claim that a deontic context activates control proactively; individuals are not just more vigilant and able to overcome any interference that may arise in the context (a sign of reactive control)—they establish a motivational mindset that is active in preventing undesirable interference.

Data from the two experiments also suggest that control is higher when the background color is red than any of the other colors. As such, we replicate results by Wang et al. (2016) and suggest that their effects are not necessarily or exclusively related to red having a deontic meaning. From the available alternative explanations for this color’s effect (see Elliot and Maier 2014), one relevant for these data is the likelihood of red prompting a motivational tendency to be alert and cautious, given that red signals danger and is associated with thoughts of failure. However, this would mean that the color red instigates an avoidance motivation, reducing performance (Maier et al. 2008). Although future studies should approach this possibility, we find no differences in the global time participants took to perform the red and the other flanker task types, as well in the accuracy of their responses (both in Experiments 1 and 2), which suggests that this alternative explanation is unlikely. Our data add support to the claim that the color red affects cognitive performance but, contrary to some studies, it is not leading to worse performance (e.g., Elliot et al. 2011; Lindsey et al. 2010). The identified better performance is likely occurring because we control our responses better when alerted by the color red (as Wang et al. 2016). However, an alternative interpretation is offered by the meta-analysis performed by Gnambs (2020) which suggests that there is great instability in the influence that is exerted by the color red over performance. The author claims that this can occur for two reasons: either because the different type of tasks vary in whether they are or not dependent on executive functions, or because the tasks may be related to different appraisals and participants may have interpreted them as a challenge or a threat (see Behnke and Kaczmarek 2018; Blascovich et al. 2004). Although these two alternative explanations should be further explored in the future, it is informative that in our first experiment, the presence of others did not moderate the effects. Previous studies suggest that the manipulation of the presence of others would impact individuals’ level of engagement, leading to patterns of challenge and threat to have a higher impact on our behavior (Blascovich et al. 1999; Fonseca et al 2014).

There are two methodological features of Experiments 1 and 2 that should be carefully analyzed in future replications in order to better understand the role of deontic over cognitive control. One relates to the order in which participants perform the tasks, since different types of carry-over effects are likely to occur. Performance on a second task will always be likely impacted by the deontic vs. non-deontic nature of the first task. In Experiment 1, only the neutral flanker task, which preceded the two deontic tasks, was protected from order effects. Although evidence did not suggest the existence of order effects within the two counterbalanced deontic flankers (prohibition and obligation), this may be because they were both deontic tasks. However, participants could be better at preventing interference in those tasks because they were better adapted to the conflict. In Experiment 2, order effects were less prone to interfere with the testing of our hypothesis, likely because it was controlled between participants. However, the “control” flanker type condition (the green color), instead of being the first, was now the last to be performed. Participants generally perform better on that task in both experimental priming conditions. This suggests some learning (adaption to conflict mechanism) even if we find no evidence of conflict adaptation across blocks. As such, future studies may consider fully counterbalancing all conditions, even taking into consideration large spillover effects (i.e., if a deontic task by itself primes control for all subsequent tasks).

The second feature to be carefully analyzed is that our claims concerning control rely only on the operationalization of a deontic context using traffic signs, and on its comparison with the gaming console controller effectively isolating the deontic component. There is no doubt that the first context is more deontic than the last, since it is a context where *must* and *ought* are activated, whereas the other defines a context of *options*. However, and especially regarding the red sign, it may, even in the console context, be understood as a communication of stopping and prohibition, equally activating control over their action through a deontic pathway. Traffic signs may isolate a specific type of deontic context where our lives may be at stake, and as such are not representative of other deontic contexts. The relevance of the specific context for our results is suggested by the mental models (Johnson-Laird et al. submitted; Oaksford and Chater 1996 for reviews) approach to deontic reasoning (see Vargas et al 2011)*.* Those studies call our attention to the fact that prohibition and obligation injunctions are represented in different ways, dependent upon the specific deontic context in question (Bucciarelli and Johnson-Laird 2005). Obligations are salient in permissible situations and prohibitions in impermissible situations. As such, the traffic sign context may have been relevant to define the pattern of results obtained in our data. We believe, therefore, that the general hypothesis that deontic context leads to proactive control must be further approached in contexts of practical significance such as morality, law, and other social norms, which may or not be related to our security systems.

We believe that our approach asks for data showing the generality of the effect in other deontic contexts, and likely ones with more ecological validity. However, we also believe that at the same time, it is relevant to continue to approach the effect with a task that is known to directly measure executive control functions. Tasks that, like the flanker task used in our studies, allow researchers to define a deontic context with deontic meaning that is not useful to perform the task (the direction of the arrow of the sign is the only information useful to perform the task). As such, future studies may continue to address deontic effects with tasks such as the flanker or the Stroop tasks. In doing so, they may focus on whether the power of the deontic context is moderated by contexts that themselves induce either more reactive control—when most trials are congruent—or more proactive control—when most trials are incongruent (e.g., Gonthier et al. 2016). In addition, within the executive control tasks, future studies may explore how control is exerted over stimulus with and without a deontic nature, these being presented either as targets or as distractors. These results would help to clarify if our mind can selectively attend to the deontic nature of the signs or if it is generally influenced by a context where deontic concerns are primed.

Even considering that our study does not provide an ecological setting where our data would directly inform traffic safety and traffic design, we believe this research is relevant to that context. This set of results suggests that we all learn the color coding used in traffic design in a way that our minds are attuned to the need for increased cognitive control in its presence. A better understanding of how cognitive control supports our efficient driving may help to better predict not only the reaction to traffic signals, but also the interferences that occur while driving (e.g., overload effects, cognitive exhaustion, etc.)

## Conclusions

Society determines, in different ways, our behavior within a deontic context. Those contexts are expected to lead us to behave in one specific direction or to avoid one specific option. Here, we show that the expectation is supported by the fact that within those contexts, individuals proactively mobilize their executive control abilities, which facilitates their capacity to deal with undesirable interferences.

## Data Availability

Data can be made available by the authors upon request.
